# Different observation period of exercise training in amyotrophic lateral sclerosis patients: A meta-analysis

**DOI:** 10.3389/fneur.2022.986882

**Published:** 2022-10-21

**Authors:** Baohua Zhou, Jiajun Wei, Yanli Zhang, Yue Liu, Shuhui Shan, Shan Ye, Baohua Li, Dongsheng Fan, Yongmei Luo

**Affiliations:** ^1^Department of Neurology, Peking University Third Hospital, Beijing, China; ^2^Nursing Department, Peking University Third Hospital, Beijing, China; ^3^Hematology, Peking University Third Hospital, Beijing, China

**Keywords:** amyotrophic lateral sclerosis, exercise, functional ability, rehabilitation, meta-analysis

## Abstract

**Objective:**

The purpose of this meta-analysis was to evaluate the effect of more intensive exercise training on the functional ability of patients with amyotrophic lateral sclerosis.

**Methods:**

Randomized controlled trials on exercise training in amyotrophic lateral sclerosis patients were retrieved from PubMed, Embase, Web of Science, Cochrane Library and other databases, and meta-analysis was conducted using a fixed effect model or random effect model. Sensitivity analysis was used as a means to study heterogeneity.

**Results:**

A total of 8 randomized controlled trials involving 330 patients with amyotrophic lateral sclerosis were included in this study. The results showed that there was statistical significance in the influence of more intensive exercise training on amyotrophic lateral sclerosis Functional Rating Scale in the short term (0–4 months) and the medium term (5–8 months) (*P* < 0.05). There was no significant difference in the effect of the amyotrophic lateral sclerosis Functional Rating Scale-Revised in the short term (0–4 months) or long term (9–12 months) (*P* ≥ 0.05). In the medium term (5–8 months), there was statistical significance (*P* < 0.05). There was no significant difference in Forced vital capacity (FVC%) in the short term (0–4 months) (*P* > 0.05).

**Conclusion:**

More intensive exercise training may slow the decline in functional score of amyotrophic lateral sclerosis patients, and more studies should be carried out in the future to verify the effect of more intensive exercise training in patients with amyotrophic lateral sclerosis.

## Introduction

Amyotrophic lateral sclerosis (ALS) is a fatal motor neuron disease characterized by degenerative changes in upper and lower motor neurons (1, 2). Generally, the disease is more common in middle-aged and elderly people, and the main clinical manifestations are progressive aggravation of skeletal muscle weakness, atrophy, muscle bundle fasciculations, bulbar paralysis and pyramidal bundle sign. The median survival time after symptoms appear is approximately equal to 3 years (3). In 2016, the estimated prevalence of ALS in mainland China was 2.97 per 100,000 person-years, with an incidence rate of 1.62 per 100,000 person-years (4). According to a systematic review and meta-analysis of the prevalence and incidence of ALS published in 2020, the global prevalence of ALS is 4.42 per 100,000 person-years, and the incidence is 1.59 per 100,000 person-years, with the incidence increasing significantly each year since 1957 (5). The patient's quality of life is seriously affected by progressive muscle atrophy and loss of movement, swallowing, speaking and breathing.

In recent years, studies have pointed out that exercise intervention for ALS patients can have a therapeutic effect on neuromuscular function, thus improving the functional ability of patients and delaying the deterioration of the disease (6). As a non-drug therapy technique, exercise training can promote the plasticity of muscle fibers and nerve tissue and have antioxidant and anti-inflammatory functions, which have a positive impact on cardiovascular, respiratory, musculoskeletal, metabolic and neuroendocrine systems (7). Rahmati et al. (8) suggests that more intensive exercise training can slow the decline in FRS-R scores in ALS patients. However, Su et al. (9) showed that more intensive exercise training had no effect. Besides the small sample size of each study, the heterogeneity of duration and type of exercise training protocols, which may give raise to inconsistent results, and the guidelines (10) suggest that the effect of more intensive exercise training on ALS patients was not clearly shown. Therefore, this study divided FRS-R into short term (0–4 months), medium term (5–8 months) and long term (9–12 months) based on different observation durations of outcome indicators. Then, Meta-analysis was used to conduct subgroup analysis to evaluate the effect of more intensive exercise training on the functional ability of ALS patients according to the observed indicators in different periods. This study aims to provide new and reliable evidence for clinical practice.

## Materials and methods

### Retrieval strategy

PubMed, Embase, Web of Science, Cochrane Library and other databases were searched from database construction to 1 February 2022. A combination of subject words and free words was used to search for published systematic reviews and included references. English search words included “Amyotrophic lateral sclerosis/sclerosis, Amyotrophic Lateral Gehrig's Disease/Charcot Disease/Motor Neuron Disease, Amyotrophic Lateral Sclerosis/Lou Gehrig's Disease/ALS Amyotrophic Lateral Sclerosis/Amyotrophic Lateral Sclerosis-Parkinsonism-Dementia Complex 1/Dementia Disease/Dementia With Amyotrophic Lateral Sclerosis” “Exercises/Physical Activity/Acute Exercise^*^/Isometric Exercise^*^/Aerobic Exercise^*^/Exercise Training^*^.”

### Inclusion and exclusion criteria

**Figure 1:**
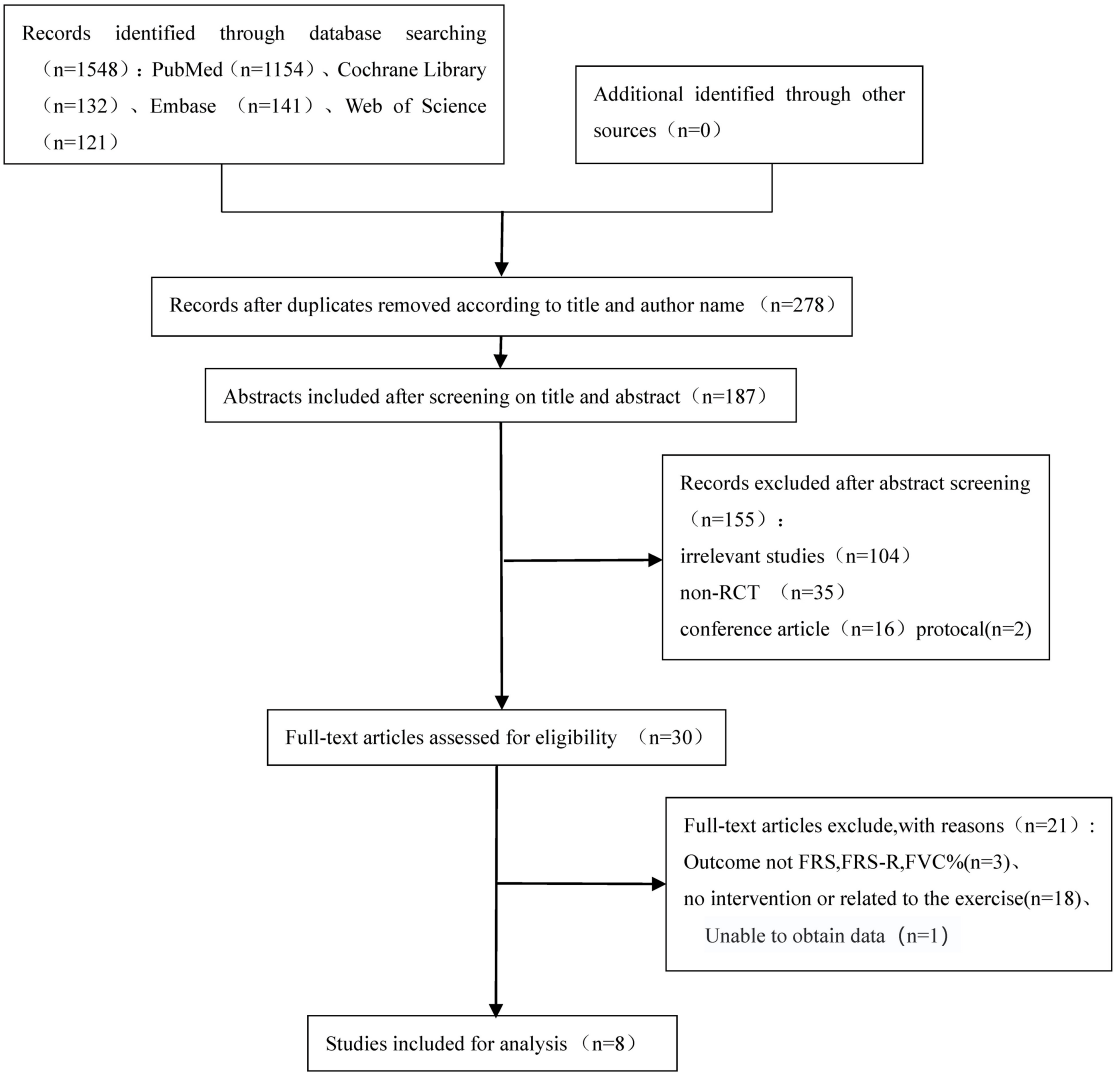
Flow diagram outlining the search.

Inclusion criteria: Study type: randomized controlled trial; Subjects: patients aged ≥ 18 years and diagnosed with ALS; Intervention measures: exercise training, including aerobic training, anaerobic training and aerobic training combined with anaerobic training; Outcome indicators: ALS Functional Rating Scale (ALSFRS), ALS Functional Rating Scale-Revised (ALSFRS-R), forced vital capacity (FVC%). Exclusion criteria: Repeated publications; The full text is still not available through various channels; Missing outcome indicators (Figure 1).

### Data extraction

Two researchers independently conducted literature screening and data extraction according to the inclusion and exclusion criteria of the literature. Duplicate literature was removed by literature management software, the title and abstract of the literature were preliminarily screened, the full text of the literature was rescreened, and the missing data were obtained by contacting the authors. In case of conflicting studies, consensus was reached through discussion or a third researcher assisted in solving the problem, and the literature was finally included. Data extraction included the following: The basic characteristics of the study, such as title, author, publication year, and research source country, were included; basic characteristics of subjects, including course of disease, sample size, etc.; intervention method and intervention time of the experimental group and control group; outcome indicators and study time points. The basic characteristics of the included studies are shown in Table 1.

**Table 1:** Characteristics of the included studies.

**Author (year)**	**Country**	**Disease duration, Mean ±SD, mos**	**Sample size**	**Intervention**	**Intervention time**	**Outcome**	**Study time point**
Kalron et al. (11)	Israel	E: 8.1 ± 12.3	16	Aerobic exercise + Stretching exercise + Resistance exercise	3 m	②③	6 w, 3 m
		C: 6.4 ± 12.2	16	Stretching exercise			
Braga et al. (12)	Portugal	E: 10.8 ± 6.5	24	Aerobic exercise	6 m	②	6 m
		C: 10.79 ± 7.7	24	Relaxation training + trunk balance + gait training			
Lunetta et al. (13)	Italy	E: 15.2 ± 7.2	30	Resistance exercise +Stretching exercise	6 m	②③	2 m, 4 m, 6 m, 6-m follow-up
		C: 13.7 ± 6.1	30	Passive exercise+ Stretching exercise			
Bello-Haas et al. (14)	Canada	E: 20.4 ± 12.8	13	Resistance exercise +Stretching exercise	6 m	①③	3 m, 6 m
		C: 15.4 ± 13.0	14	Stretching exercise			
Drory et al. (15)	Israel	E: 20.7	14	Aerobic exercise	12m		3m, 6m, 9m, 12m
		C: 19.4	11	Only usual daily life			
				Requirement, no physical			
				Activity			
Plowman et al. (16)	America	E: 20.9 ± 14.5	24	Hight intensity aerobic exercise	2 m	③	2 m
		C: 16.9 ± 6.8	24	Low intensity aerobic exercise			
Pinto et al. (17)	Portugal	E: 11.5 ± 5.3	13	Aerobic exercise, 8 m	8 m		4 m, 8 m
		C: 12.6 ± 6.6	13	The first 4 months: Low intensity aerobic exercise For the next 4 months: Aerobic exercise with the same intensity as the experimental group			
Zucchi et al. (18)	Italy	E: 15.67 ± 9.74	32	Stretching exercise + Aerobic exercise, 5 times/week	10 w	②③	3, 6, 9, 12, 18, 24-m follow-up
		C: 16.64 ± 8.98	32	Stretching exercise + Aerobic exercise, 2 times/week			

① ALS Functional Rating Scale (ALSFRS), ⁡ ALS Functional Rating Scale-Revised (ALSFRS-R), ⁢ Forced vital capacity (FVC%).

E: more intensive exercise training, C: conventional care group.

### Quality evaluation

**Figure 2:**
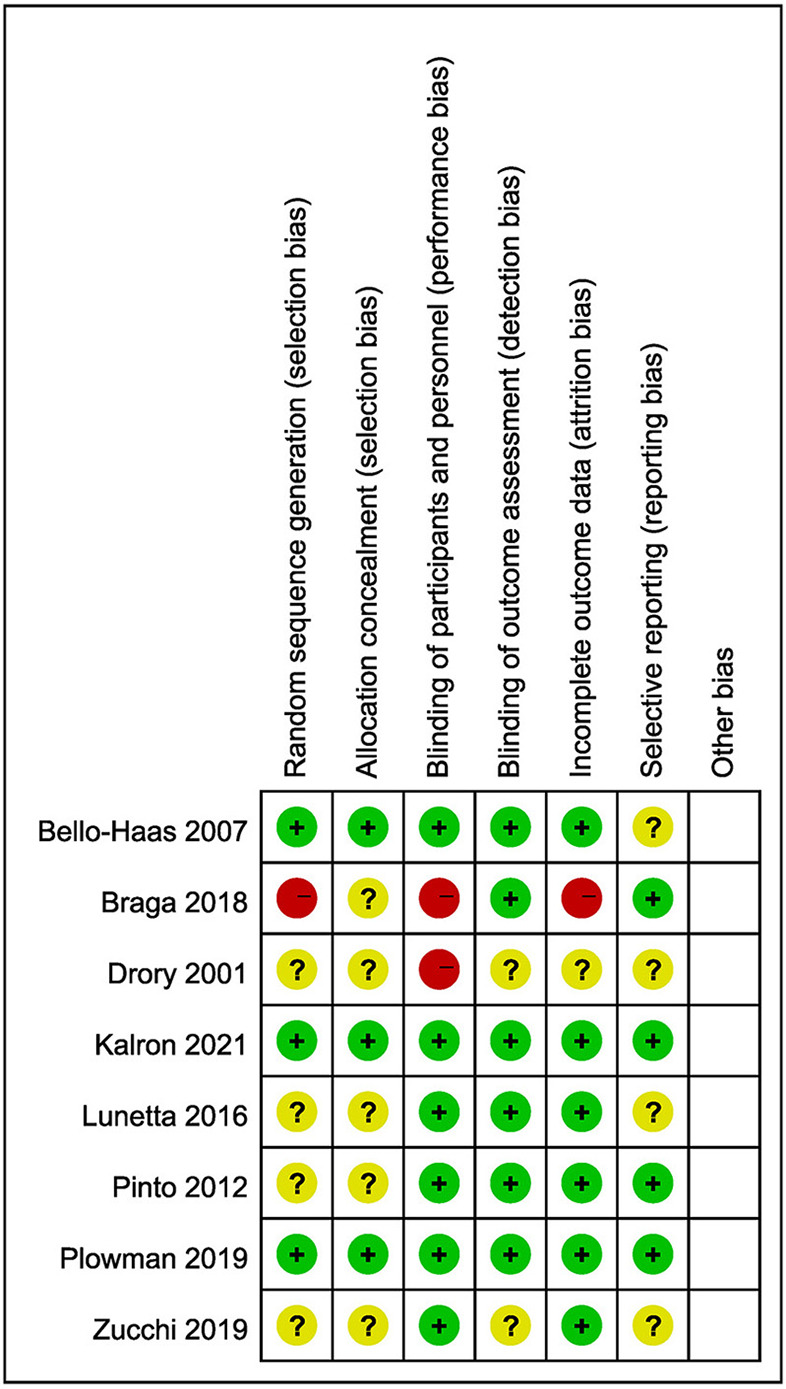
Risk of bias graph.

Two researchers used the evaluation criteria of the Cochrane Quality Evaluation Manual 5.1.0 to evaluate the quality of the included literature. The evaluation items included the following seven aspects: generation of random sequence; distribution hidden; blind method was applied to the research object and the intervention object; blind method was applied to the evaluators; data integrity of results (lost to follow-up); selective reporting results; and any other bias. Evaluators were required to make judgements of unclear, high or low risk of bias for each evaluation item. If two researchers had different opinions, a third researcher was required to intervene to solve the problem. If the above criteria are fully met, the possibility of bias is small, and the quality grade is A. If some of them meet the above criteria, the probability of bias is medium, and the quality grade is B. If the above criteria are not met completely, the possibility of bias is greater, and the quality grade is C (Figure 2).

### Data analysis

Rev Man 5.3 software was used for meta-analysis. The results were all continuous variables, and mean difference (MD) was used as effect analysis statistics, with 95% CI for each effect size. Using χ2 inspection and i.2 value analysis, heterogeneity was assessed. For example, *P* > 0.1 and I^2^ < 50% indicated no significant statistical heterogeneity, and a fixed effect model was used. *P* < 0.1 and I^2^ ≥ 50% indicated significant statistical heterogeneity, and a random effect model was adopted. *P* < 0.05 was considered as statistically significant.

## Results

### The results of the literature search and the basic characteristics of the included studies

A preliminary search was conducted to obtain 1,548 studies, with the remaining 278 studies removed. According to the inclusion and exclusion criteria, the titles and abstracts of the literature were read, the full text of the excluded literature was read, and 8 English studies were finally included. The literature screening process is shown in **Figure 4**. Included articles were published between 2001 and 2021, and two were from Italy (13, 18), 2 were from Israel (11, 15), 2 were from Portugal (12, 17), 1 was from the United States (16), and 1 was from Canada (14). A total of 330 subjects were included, with 166 in the experimental group and 164 in the control group.

### Evaluation of methodological quality

A total of 8 literatures were included in this study, four (11, 12, 14, 16) of which introduced the generation of random sequence. The literature introduces the generation of random sequence. Three of the article (11, 14, 16) methods of allocation hiding are reported, 2 studies (11, 14) used sealed envelopes form, 1 paper (16) use random schedule form. All the literature described the situation and causes of loss to follow-up, the baseline data of the experimental group and the control group were compared, and the results showed no statistical significance (*P* > 0.05). In two articles (11, 16), the literature quality was grade A; therefore, the literature included in this study was of high quality, and the evidence credibility was high. The methodological quality evaluation of the literature is shown in Table 2.

**Table 2:** Methodological quality evaluation of the included studies.

**Study**	**Selection bias**		**Performance bias**	**Detection bias**	**Attrition bias**	**Reporting bias**	**Other bias**	**Grade**
	**Random sequence**	**Allocation concealment**						
Kalron et al. (11)	Low risk	Low risk	Low risk	Low risk	Low risk	Low risk	No	A
Braga et al. (12)	High risk	Unclear	High risk	Low risk	High risk	Low risk	No	B
Lunetta et al. (13)	Unclear	Low risk	Unclear	Unclear	Low risk	Unclear	No	B
Bello-Haas et al. (14)	Low risk	Low risk	Unclear	Low risk	Low risk	Unclear	No	B
Drory et al. (15)	Unclear	Low risk	High risk	Unclear	Unclear	Unclear	No	B
Plowman et al. (16)	Low risk	Low risk	Low risk	Low risk	Low risk	Low risk	No	A
Pinto et al. (17)	Unclear	Low risk	Unclear	Low risk	Low risk	Low risk	No	B
Zucchi et al. (18)	Unclear	Low risk	Unclear	Unclear	Low risk	Unclear	No	B

### Effect on functional scores in patients with amyotrophic lateral sclerosis

**Figure 3:**
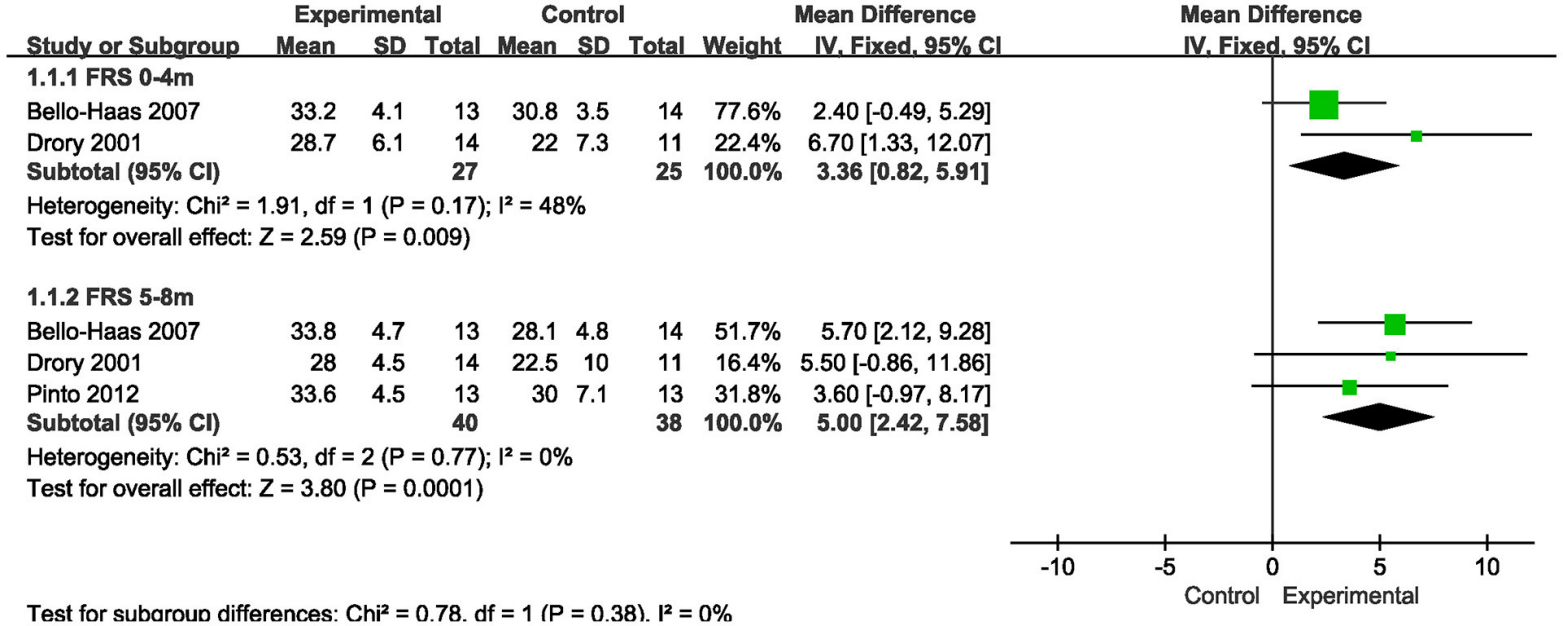
Forest plot of experimental group vs. control group. Outcome: ALSFRS.

There were two studies (14–16) where measures of FRS observed in the short term (0–4 months) were reported, and results showed the decline was slower in the more intensive exercise training group compared with conventional care group [MD = 2.40, 95% CI (0.23, 4.58), *P* = 0.03]. In addition, there were 3 studies (14, 15, 17) where indicators observed during the medium-term period (5–8 months) of FRS were reported, showing that the decline was slower in the more intensive exercise training group than conventional care group [MD = 5.00, 95% CI (2.42, 7.58), *P* = 0.0001]. The results are shown in Figure 3.

**Figure 4:**
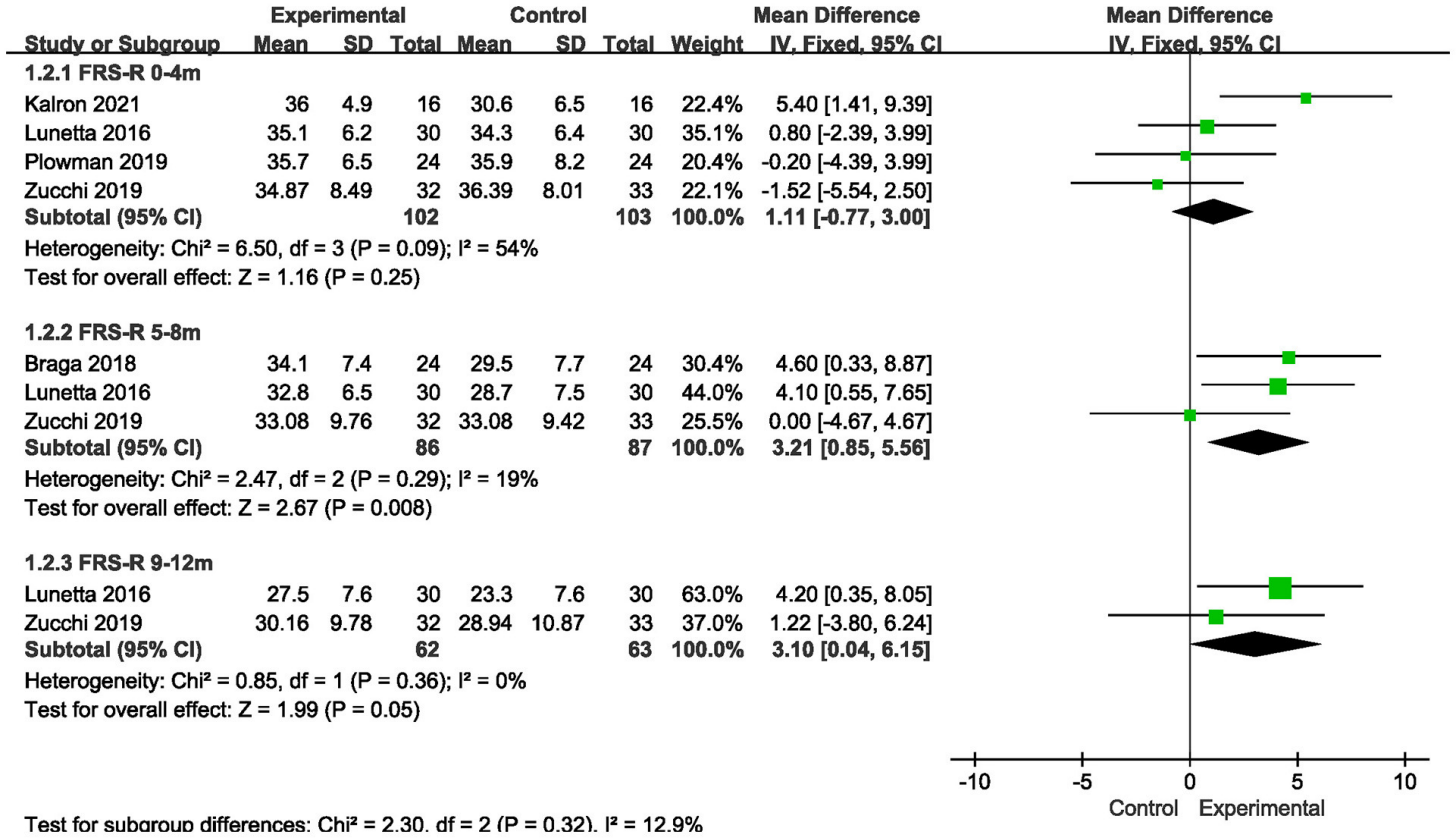
Forest plot of experimental group vs. control group. Outcome: ALSFRS-R.

There were 4 studies (11, 13, 16, 18) where measures observed in FRS-R over a short-term period (0–4 months) were reported, there was no statistically significant difference between the more intensive exercise training group and the conventional care group on the short-term (0–4 months) ALSFRS-R score scale [MD = 1.11, 95% CI (−0.77, 3.00), *P* = 0.25]. In addition, there were 3 studies (12, 13, 18) whose results showed statistically significant differences in ALSFRS-R scores between the more intensive exercise training group and the conventional care group during the medium-term period (5-8 months) [MD = 3.21, 95% CI (0.85, 5.56), *P* = 0.008]. In addition, there were two studies (13, 18) where indicators of FRS-R observed over a long-term period (9–12 months) were reported, and meta-analysis showed no difference between the more intensive exercise training group and the conventional care group. The results are shown in Figure 4.

### Effects on respiratory function in patients with amyotrophic lateral sclerosis

**Figure 5:**
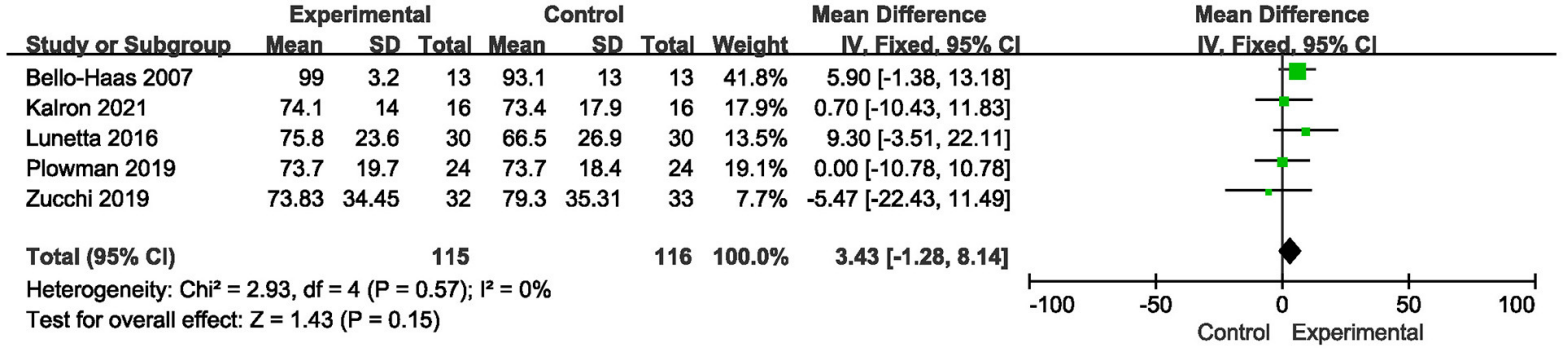
Forest plot of experimental group vs. control group. Outcome: FVC%.

There were 5 studies (11, 13, 16, 18) where the prognostic percentage of forced vital capacity was reported over a short period of time (0–4 months). The results showed no significant difference in FVC% between the more intensive exercise training group and the conventional care group [MD = 3.43, 95% CI (−1.28, 8.14), *P* = 0.15]. The results are shown in Figure 5.

## Discussion

In this meta-analysis, more intensive exercise training slowed the decline in FRS observed in the short term (0–4 months) and the medium term (5–8 months). For FRS-R, there was no slowdown in short-term (0–4 months) and long-term (9–12 months) data results, but the decline slowed significantly in medium-term (5–8 months) observed indicators. For FVC%, the short-term (0–4 months) observation showed no slowdown.

The results of this study showed significant slowdown in both FRS and FRS-R scores observed during the medium term (5–8 months). A large body of data suggests that mitochondrial damage leads to neuronal failure, which leads to severe muscle paralysis (19), while exercise may slow the degeneration of motor neurons, reduce oxidative stress on fast-contracting type II muscle fibers and help them transform into type I muscle fibers in muscles, making them more resistant to fatigue (20). On the other hand, exercise increases glucose utilization, which contributes to improved work performance and daily activities, and increased mitochondrial biogenesis enhances antioxidant defenses. Regular physical exercise may lead to increased muscle mass and induce regeneration by activating astrocytes, a process that may be altered in ALS (21), and could have possibly slow the decline in the observation index over the medium term (5–8 months) of the FRS score and FRS-R score. For the indicators observed in the long term (9–12 months), due to the lack of relevant data of the FRS score, the FRS-R score of this meta-analysis, although *P* = 0.05, showed no significance. However, since only two studies were included and the sample size was small, it may have resulted in no effect on the indicators observed in the long term (9–12 months), in addition, overall a slight positive difference toward the intervention arm is detected, though not at a significant level, thus this finding needs to be further studied in expanded sample sizes.

However, for the FRS score and FRS-R score observed in the short term (0–4 months), we obtained different results, where the FRS score declined more slowly but the FRS-R score did not. The reason for this difference may be related to the fact that respiratory function is not significantly affected in the early stage of the disease. The ALSFRS-R adds the content related to breathing, sitting breathing and respiratory insufficiency, compared with the ALSFRS, while respiratory dysfunction appears months to years after diagnosis in ALS patients (22). In general, patients with medulla oblongata and thoracic medulla develop dyspnoea earlier than those with cervical and lumbar medulla. A total of 8 randomized controlled trials were included in this study. The disease course of the subjects was mostly within 20 months, and they were in the early stage of the disease with no obvious respiratory function involvement. Besides, a possible selection bias of all the studies is exercise training in patients with respiratory involvement is less likely to be offered due to the easy fatigue and lack of endurance of these patients to the majority of exercise protocols. These may have resulted in no slowdown in ALSFRS-R scores in the short term (0–4 months).

To explore the influence of respiratory function on the difference between FRS and FRS-R in the short term (0–4 months), the index FVC% was used in the pulmonary function in this meta-analysis. However, since there were only a few studies and the data were only observational indicators in the short term (0–4 months), the results showed no significance. However, the structural and functional characteristics of respiratory muscle fibers are not fixed and can be changed according to training (23), so whether FVC% can produce benefits in the medium and long term needs to be confirmed by multicentre studies with large sample sizes in the future.

This study is the first to report the effect of more intensive exercise training on FRS-R observation indicators in ALS patients in the long term (9–12 months), and the results showed no statistical significance. It is speculated that the reason for this result may be related to the small number of included studies and sample size. Therefore, long-term studies with large sample sizes are still needed in the future to confirm the effect of more intensive exercise training on patients' functional ability. Furthermore, an aspect that would be sound to be included into discussion is the role of eventual exercise-related disease biomarkers that could be useful to combine with clinical outcome measures in order to increase significance of exercise training interventions in ALS (24).

## Strengths and limitations

Advantages: The literature was screened in strict accordance with the inclusion and exclusion criteria. The 9 included studies were from Italy, Israel, Portugal, the United States, the Netherlands and Canada. This was a meta-analysis of the more intensive exercise training of ALS patients with a large number of included studies, and the literature was updated as of 1 February 2022. The meta-analysis included the long-term effects of more intensive exercise training on the functional ability of ALS patients (9–12 months) for the first time, and the results further enriched the effects of more intensive exercise training. Each study procedure was completed by two researchers independently, and the procedures and procedures in the Cochrane Systematic Review Manual V 5.1.0 were strictly followed using rigorous methodology. Limitations: First, the sample data of some analyses are too small, which may affect the accuracy of the results and needs to be confirmed by more studies. Second, it is difficult to double blind due to intervention limitations, but it is suggested that data collectors may be blinded in the future. Third, some of the results are relatively heterogeneous, which may be related to different exercise training methods. Different exercise methods may also bring differences in the improvement of motor function, and can be investigated in future research studies. Fourth, in the definition of the case history of exercise therapy in the selected studies did not address the subtype of ALS or the velocity of its progression rate, an aspect that needs to be taken into consideration in the future. In addition, there is a additional sources of bias, the heterogeneity of duration of exercise protocols, which may affect the interpretation of results: while some studies intervened for longer periods (10–12 months), other just for short periods (2–3 months); however, the clinical outcomes were measured at standardized time points, which for some studies fell within the intervention period while for others just in a follow-up period. This issue should be taken into consideration when looking at early, middle or long-term effects: overall, a beneficial effects might be observed while continuing exercise training or after some discontinuation.

## Conclusion

The meta-analysis of this study showed that more intensive exercise training could slow down the decline in functional scores of ALS patients in the short term (0–4 months) and the medium term (5–8 months). Therefore, it might be that ALS patients persist in more intensive exercise training in clinical practice to improve their functional ability. In the future, more long-term large-sample studies should be carried out to verify the effectiveness of exercise training in ALS patients.

## Data availability statement

The original contributions presented in the study are included in the article/supplementary material, further inquiries can be directed to the corresponding author.

## Author contributions

BZ and YZ screened the articles. SS and BZ extracted and cross-checked the data. JW and YLi participated in the statistical analysis. JW wrote the first draft of the manuscript. SY, BL, DF, and YLu revised and discussed the final version. All authors have read and approved the final version of the manuscript.

## Conflict of interest

The authors declare that the research was conducted in the absence of any commercial or financial relationships that could be construed as a potential conflict of interest.

## Publisher's note

All claims expressed in this article are solely those of the authors and do not necessarily represent those of their affiliated organizations, or those of the publisher, the editors and the reviewers. Any product that may be evaluated in this article, or claim that may be made by its manufacturer, is not guaranteed or endorsed by the publisher.
